# Iron-Storage Disorder Presenting as Chronic Diarrhea

**DOI:** 10.7759/cureus.18864

**Published:** 2021-10-18

**Authors:** Nikitha Vobugari, Jeffrey Kim, Kejal D Gandhi, Zone-En Lee, Hedy P Smith

**Affiliations:** 1 Internal medicine, MedStar Washington Hospital Center, Washington, D.C., USA; 2 Gastroenterology and Hepatology, MedStar Georgetown University Hospital, Washington, D.C., USA; 3 Internal Medicine, MedStar Washington Hospital Center, Washington, D.C., USA; 4 Hematology and Medical Oncology, MedStar Washington Hospital Center, Washington, D.C., USA

**Keywords:** pancreatic iron overload, chronic diarrhea, hemochromatosis, iron storage disorder, pancreatic exocrine insufficiency

## Abstract

The involvement of the endocrine pancreas leading to bronze diabetes is well studied. However, little is known about the pathophysiology of iron dysregulation involving the exocrine pancreas. We present a unique association between the exocrine pancreas and iron dysregulation. A 45-year-old female presented with chronic diarrhea and low fecal elastase indicative of pancreatic exocrine dysfunction. MRI of the abdomen/pelvis showed iron deposition in the pancreas, suggesting an associated iron-storage disorder without features suggesting chronic pancreatitis. Association of an iron-storage disorder with pancreatic exocrine dysfunction has been reported only in one other case report. Pancreatic exocrine dysfunction can be directly associated with an iron-storage disorder that involves the pancreas. This should be included in the differential and diagnostic work-up of chronic diarrhea of unclear etiology. Based on the literature, we have highlighted the potential pathophysiology relevant to the case.

## Introduction

Chronic diarrhea (CD) is prevalent in 2-3% of the population and has a broad differential diagnosis. Iron-storage disorders (ISD) causing pancreatic exocrine dysfunction (PED) are overlooked as a cause of CD, given the more common presentation with liver function abnormalities and liver failure, skin hyperpigmentation, diabetes mellitus, and arthralgias. Iron deposition in pancreatic beta cells is a well-established cause of diabetes mellitus in patients with hemochromatosis [1]. The association between iron dysregulation and PED has been reported as early as 1965, but this interrelation and its mechanisms were not well acknowledged [2-4]. We report a case of a CD with PED and iron deposition in the pancreas.

## Case presentation

Our patient was a 45-year-old African American female with a past medical history of pre-diabetes mellitus, hypertension, alcoholic hepatitis, cholecystectomy, and anemia requiring multiple (10-15) blood transfusions over the span of 3-4 years. She presented with daily non-bloody, greasy diarrhea, including nocturnal episodes. She reported associated crampy abdominal pain and extreme fatigue.

**Figure 1:**
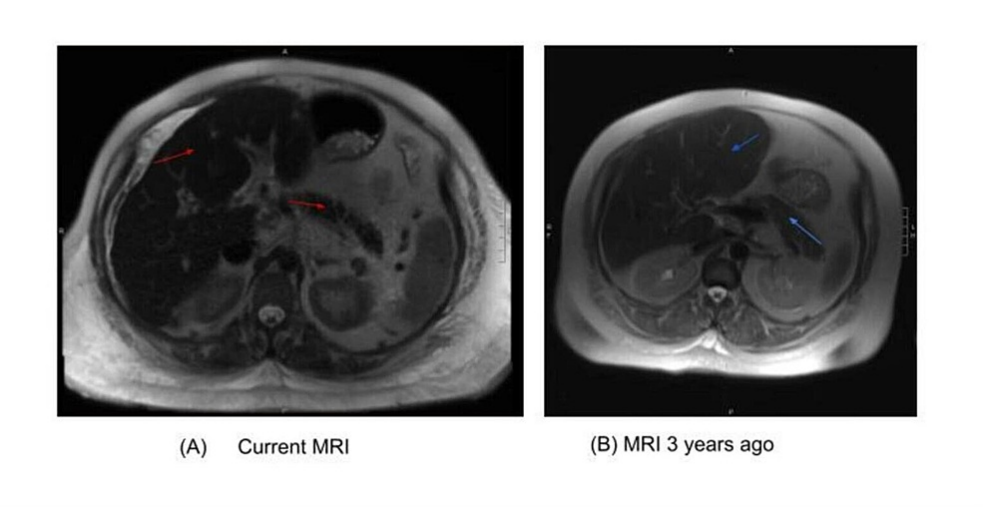
Comparison of Contrast-enhanced MRI-Axial HASTE section: Iron deposition depicted by dark black color in liver and pancreas - red arrows in image A vs. normal liver and pancreas 3 years ago depicted by grey color in liver and pancreas - blue arrows in image B.

Testing was consistent with secretory diarrhea with stool osmotic gap 33 mOSm/kg, PED with stool pancreatic elastase <15 mcg/g, mild elevation in liver transaminases with impaired hepatic synthetic function with INR of 1.2 and hypoalbuminemia 1.2 g/dl. Infectious etiologies, inflammatory bowel disease, autoimmune disorders, celiac disease, and neuroendocrine causes were ruled out (Table 1). Contrast-enhanced MRI of Abdomen and Pelvis with Axial T2 HASTE sequence revealed low signal absorption in the liver and pancreas, indicating iron deposition, chronic hepatic steatosis, and cirrhotic changes and did not reveal typical radiological findings of alcoholic chronic pancreatitis (Figure 1). MRI three years prior did not show iron deposition (normal greyish color of liver and pancreas) and no pancreatic abnormalities. Hemoglobin/ hematocrit was 9.8 g/dl / 22.2% with an MCV of 90 (current admission). Serum iron level was 83 mcg/dl, transferrin saturation (TSAT) 120%, ferritin 2442 ng/ml, which were consistent findings over three years of retrospective chart review (Table 2). HFE gene mutation analysis revealed H63D heterozygosity as the only mutation. There was no family history of hemochromatosis. Her blood glucose ranged between 90-120s mg/dl and hemoglobin A1C 6.3%. An echocardiogram showed the left ventricle normal in size, shape, and thickness with an ejection fraction of 55-60%, though the CT chest reported heart enlargement with LV chamber dilation.

**Table 1:** Relevant laboratory findings

Test		Value/ Result
Stool osmotic gap (mOsm/kg)		33
Stool pancreatic elastase (mcg/g)		<15
Serum Lipase (U/L)		58
AST (U/L)		47
ALT (U/L)		14
ALP (U/L)		68
T. Bilirubin (mg/dL)		0.3
Platelet count (k/ul)		115
INR		1.2
Albumin (gm/dL)		1.2
Hb (gm/dL)		9.8
Hct(%)		22.2
MCV(FL)		90
Iron (mcg/dL)		83
TSAT (%)		120
Ferritin (ng/ml)		2442
Molecular pathology for Hereditary Hemochromatosis		HFE H63D: Heterozygous HFE C282Y: Negative HFE S65C: Negative
Hemoglobin electrophoresis		Hemoglobin A (97.3%) and A2 (2.7%).
Random blood glucose (mg/dl)		90-120
Hemoglobin A1C (%)		6.3
TSH (uIU/mL)		3.23
Serum gastrin level (pg/mL)		32
AM Cortisol (mcg/dL)		6.7
Urine 5-HIAA/Creatinine		2
Serum vasoactive peptide (pg/mL)		<13
Serum chromogranin A (ng/mL)		241
Celiac work up	tTG IgA (U/mL)	2
	tTG IgG (U/mL)	7
	Serum IgA(mg/dL)	930
Stool leukocytes		None seen
Stool Ova & Parasites		None seen
Stool Clostridium difficile antigen		Negative
Stool BioFire (Campylobacter, Plesiomonas shigelloides, Salmonella, Vibrio, Yersinia, Enteroaggregative E.Coli, Enteropathogenic E. Coli, Enterotoxigenic E.Coli, Shiga toxin-producing E.Coli, Shigella/Enteroinvasive E.Coli, Cryptosporidium, Cyclospora cayetanensis, Entamoeba histolytica, Giardia, Adenovirus F40/4, Astrovirus, Norovirus GIGII, Rotavirus, Sapovirus		Negative
HIV1/0/2 Ab/Ag		Non-Reactive
Hepatitis Panel		Negative
Urine Beta HCG		Negative
Fecal Calprotectin (mcg/gm)		26
Colonoscopy with multiple biopsies		Normal architecture and mild chronic inflammation. No evidence of active inflammation, microscopic colitis, granulomas, dysplasia, parasites, or viral inclusions

Given PED, the patient was started on pancrelipase dosed at 75,000 units with meals and 48,000 units with snacks. The patient's diarrhea resolved over several days. Hematology service was consulted for hemochromatosis and for the need for phlebotomy or chelation therapy. Other causes for iron overload were considered, including genetic mutations or polymorphisms in the ferroportin gene (prevalent in African American population), iron overload due to sideroblastic anemia related to chronic alcoholism, iatrogenic iron overload due to chronic transfusions. Unfortunately, the patient was lost to follow up, and work up including bone marrow biopsy could not be completed.

**Table 2:** Iron studies over three years

	02/05/2020	01/18/2020	03/2019	12/2017
Iron (mcg/dl)	59	53	83	172
TIBC	114	45	69	172
iron sat (%)		118	120	100
Transferrin (mg/dl)	<80	44	60	132
Ferritin (ng/ml)	947	1245	2442	1440

## Discussion

We present a case of chronic diarrhea due to pancreatic insufficiency caused by pancreatic iron overload. ISD was diagnosed based on MRI findings of iron deposition in the liver and pancreas and laboratory findings of elevated serum ferritin and transferrin saturation. MRI revealed cirrhotic liver changes, which could be from chronic alcoholism, iron deposition, or both. However, it did not reveal features typical for chronic pancreatitis from chronic alcohol use, such as pancreatic calcifications, pancreatic duct dilatation, or beading, absence of which made our suspicion of ISD as the more likely cause of PED [5].

Iron overload and iron storage disorders (ISD) have several causes, including hereditary hemochromatosis due to mutations in the HFE, ferroportin and other iron regulatory genes, transfusion-related iron overload, and rare disorders such as sideroblastic anemia. ISD, in our case, is likely multifactorial, including heterozygosity for the H63D HFE gene mutation, multiple blood transfusions, alcoholism, and undiagnosed ferroportin gene mutation (autosomal dominant mutation in the African American population) [4,6,7].

To the best of our knowledge, there is only one other reported case of hemochromatosis causing acute PED following a viral infection by Jansen et al. in the 1980s [8]. Our case is unique as we report a symptomatic PED (proven low stool pancreatic elastase) due to iron deposition in the parenchyma. This report highlights that iron deposition is not exclusive to the beta-pancreatic acinar cells, but can also accumulate in the exocrine cells causing pancreatic enzyme deficiency [1,9,10]. In a study of 32 hereditary hemochromatoses (HH) patients with pancreatic insufficiency, 10 patients were treated with venesections compared to 22 untreated patients. When both these groups were treated with secretin and cholecystokinin, a significantly low concentration of pancreatic enzymes in the duodenal aspirate was found in untreated hereditary hemochromatosis compared to those who were treated with venesections, implying a direct association of iron overload and PED. This supports the finding in our case [11].

The precise mechanism of PED in ISD is still unclear and needs to be further investigated. Over the past two decades, with several developments in iron metabolism and exocrine pancreas, few studies have delved into this association with some evidence of this crosstalk. In one murine study, the introduction of point mutation (C326S) preventing hepcidin-mediated-ferroportin control was shown to induce severe iron overload and fatal pancreatic exocrine failure apparent as decreased pancreatic elastase [12]. Another murine study highlighted the pancreas as a labile organ for iron deposition similar to the liver and heart with an efficient iron efflux mechanism with hephaestin and ceruloplasmin carriers. A disruption in these iron efflux carriers and channels can potentially cause exocrine iron deposition [13]. Increased iron levels leading to oxidative stress have also been recognized to affect pancreatic acinar cells and may cause PED [14].

In contrast, other studies have shown iron dysregulation with increased ferritin and reduced hepcidin caused by inflammation from pancreatitis with alterations in circulating markers of iron absorption and intrapancreatic iron deposition in acute or chronic pancreatitis [2,3,15]. It is also important to note the possible confounding effects of concurrent liver disease causing increased ferritin and reduced hepcidin in these patients. In our patient, iron studies were consistent with ISD existing for several years, with subsequent MRI abdomen series showing progressive iron deposition over three years. Subsequently, the patient suffered chronic diarrhea and evidence of PED by low fecal elastase which improved with pancreatic enzyme supplementation. Therefore, we consider that ISD leading to PED is likely in our patient.

Early treatment of HH has shown improvement in iron deposition in the liver, heart, and skin, while DM, arthropathy, and hypogonadism are often irreversible [16]. Further studies are indicated to see if treatment with phlebotomy or iron chelation therapy earlier in ISD can prevent PED occurrence or progression of PED.

## Conclusions

The pancreas is concealed by extensive studies on the effects of iron metabolism and the liver. The involvement of the pancreas is thought to be limited to bronze diabetes in ISD. It is prudent to identify the direct association of PED and ISD that involves the pancreas and should be included in the differential and diagnostic work-up of chronic diarrhea of unclear etiology. This case also supports a need for prospective studies to evaluate this association.
